# Coronary Spasm Testing with Acetylcholine: A Powerful Tool for a Personalized Therapy of Coronary Vasomotor Disorders

**DOI:** 10.3390/life14030292

**Published:** 2024-02-22

**Authors:** Filippo Luca Gurgoglione, Luigi Vignali, Rocco Antonio Montone, Riccardo Rinaldi, Giorgio Benatti, Emilia Solinas, Antonio Maria Leone, Domenico Galante, Gianluca Campo, Simone Biscaglia, Italo Porto, Stefano Benenati, Giampaolo Niccoli

**Affiliations:** 1 Division of Cardiology, Parma University Hospital, University of Parma, 43126 Parma, Italy; filippolucagurgoglione@gmail.com; 2 Division of Cardiology, Parma University Hospital, 43126 Parma, Italy; luvignali@ao.pr.it (L.V.); giorgiobenatti88@gmail.com (G.B.); esolinas@ao.pr.it (E.S.); 3 Department of Cardiovascular Sciences, Fondazione Policlinico Universitario A. Gemelli IRCCS, 00168 Rome, Italy; rocco.montone@gmail.com; 4 Department of Cardiovascular and Pulmonary Sciences, Catholic University of the Sacred Heart, 00168 Rome, Italy; rinaldi.riccardo92@gmail.com; 5 Center of Excellence in Cardiovascular Sciences, Ospedale Isola Tiberina, Gemelli Isola Roma, 00186 Rome, Italy; antoniomarialeone@gmail.com (A.M.L.); domenico.galante1991@gmail.com (D.G.); 6 Cardiology Unit, Azienda Ospedaliero Universitaria di Ferrara, 44124 Ferrara, Italy; cmpglc@unife.it (G.C.); bscsmn@unife.it (S.B.); 7 Department of Internal Medicine, University of Genoa, 16126 Genoa, Italy; italo.porto@unige.it (I.P.); stefanobenenatimd@gmail.com (S.B.); 8 Cardiovascular Disease Unit, IRCCS Ospedale Policlinico San Martino—Italian IRCCS Cardiology Network, 16126 Genoa, Italy

**Keywords:** coronary vasomotor disorders, intracoronary provocative testing, coronary microvascular dysfunction, personalized therapy

## Abstract

Coronary vasomotor disorders (CVD) are characterized by transient hypercontraction of coronary vascular smooth muscle cells, leading to hypercontraction of epicardial and/or microvascular coronary circulation. CVDs play a relevant role in the pathogenesis of ischemia, angina and myocardial infarction with non-obstructive coronary arteries. Invasive provocative testing with intracoronary Acetylcholine (ACh) administration is the gold standard tool for addressing CVD, providing relevant therapeutic and prognostic implications. However, safety concerns preclude the widespread incorporation of the ACh test into clinical practice. The purpose of this review is to shed light on the pathophysiology underlying CVD and on the clinical role of the ACh test, focusing on safety profile and prognostic implications. We will also discuss contemporary evidence on the management of CVD and the role of the ACh test in driving a personalized approach of patients with CVD.

## 1. Introduction

Ischemia (INOCA) [1], angina (ANOCA) [2] and myocardial infarction (MINOCA) [3] with non-obstructive coronary artery disease are increasingly recognized subsets of ischemic heart disease. INOCA accounts for nearly half of coronary angiographies performed due to suspected myocardial ischemia [4], while MINOCA accounts for up to 10% of patients presenting with acute MI [3].

**Figure 1:**
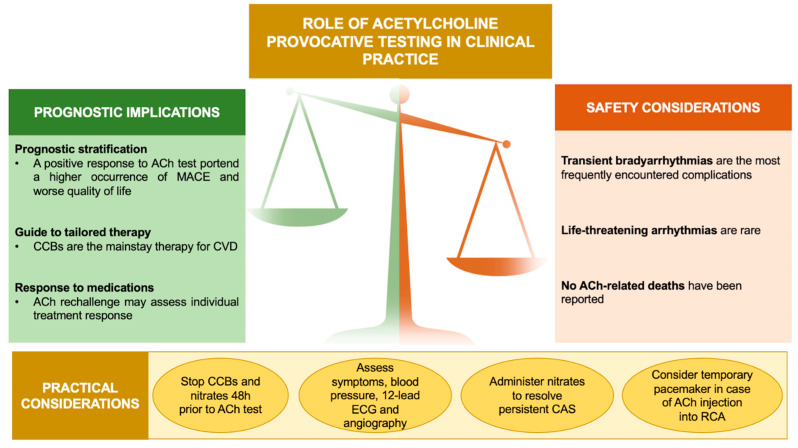
Role of ACh provocative testing in clinical practice. Abbreviations: ACh: acetylcholine; CAS: coronary artery spasm; CCBs: calcium channel blockers; CVD: coronary vasomotor disorders; ECG: electrocardiogram; MACE: major adverse cardiovascular events; RCA: right coronary artery.

Coronary vasomotor disorders (CVD), caused by a transient hypercontraction of epicardial and/or microvascular coronary circulation, play a relevant role in the pathogenesis of INOCA/ANOCA and MINOCA [5,6,7,8,9]. Invasive provocative testing with Acetylcholine (ACh) is the gold standard for the diagnosis of coronary artery spasm (CAS) [10,11,12], albeit safety concerns preclude its widespread incorporation into routine clinical practice. In this respect, it is worth noting that there is growing evidence for the relative safety of ACh-induced side effects [13,14,15,16], while emphasizing its relevant therapeutic and prognostic implications [6,7,17,18] (Figure 1).

In this review, we will provide an overview of CVD and dive into the clinical role of the ACh test. Furthermore, we will outline contemporary evidence on targeted therapy of CVD and discuss possible future perspectives.

## 2. Epidemiology

The prevalence of CVD remains unclear. The lack of awareness about coronary spasm together with the underuse of the ACh test contribute to this uncertainty. The pivotal ACOVA (Abnormal COronary VAsomotion in patients with stable angina and unobstructed coronary arteries) study identified CVD as a pathophysiological mechanism of ANOCA more than ten years ago: among a cohort of 124 patients who underwent an ACh test, CAS was elicited in 62% of patients (45% epicardial CAS, 55% microvascular CAS) [6]. Consistent with this, Montone et al. found a positive response to an ACh test in 58.4% of patients, with a higher prevalence of epicardial compared to microvascular CAS (64.3% vs. 35.7%) [7], and a large meta-analysis by Mileva et al. documented CAS in 62% of 14,427 patients with non-obstructive coronary artery disease (NOCAD) [19]. In keeping with these findings, the overall incidence was 43% for epicardial CAS and 25% for the microvascular endotype in a meta-analysis by Woudstra et al. [20]. Conversely, in the study by Kornst et al., CVDs were present in 83.8% of patients with ANOCA [21].

Less data is available with respect to MINOCA. Montone et al. found that CAS was present in about 53.7% of patients with a diagnosis of MINOCA [15]. Similarly, the rate of positive ACh test was 49% in the CASPAR (Coronary Artery Spasm as a Frequent Cause for Acute Coronary Syndrome) study [22] and 45% in the study by Deyama et al. [23]. Nakayama et al. found a high prevalence of Ach-induced CAS (79.2%) among 221 patients with MINOCA without a culprit lesion [24]; in contrast, only 3.7% of MINOCA presentations were attributable to CAS in the VIRGO study [25].

Differences in invasive provocative test protocols and patient selection might account for the high heterogeneity between studies. Contemporary research supports the hypothesis that the occurrence of CAS is dependent on ACh doses and injection speed [20,21,22,23,24,25,26,27]. Moreover, while a coronary constriction >90% is required to establish epicardial spasm according to the Coronary Vasomotion Disorders International Study (COVADIS) criteria [11], in some studies, epicardial spasm was diagnosed in case of coronary constriction >50%.

It is worth noting that Asian patients are more susceptible to CAS compared to Western subjects [6,28]: some genetic polymorphisms predisposing to CAS may be implicated [29,30,31]. Furthermore, epicardial spasm occurs more frequently in men [17,32], who are characterized by a higher prevalence of smoking habit and non-obstructive CAD, while women are more likely to develop microvascular CAS and suffer from coronary microvascular dysfunction (CMD) [33,34,35].

## 3. Pathophysiology

Two distinct pathological conditions may contribute to CAS: firstly, the presence of endothelial dysfunction, and secondly, the evidence of increased reactivity of vascular smooth muscle cells (VSMCs).

### 3.1. Endothelial Dysfunction

The endothelium, a monolayer of cells located in the intima on the luminal side of the vessels, orchestrates several pathways implicated in vascular homeostasis [36]. Hemodynamic (variations in blood pressure and flow) and metabolic stimuli, together with nervous autonomic system and circulating vasoactive substances, drive the endothelial synthesis of nitric oxide (NO), in order to modulate coronary artery tone. In detail, NO is able to diffuse into VSMCs, inducing the synthesis of cyclic guanosine monophosphate and ultimately resulting in coronary vasodilation [37]. Notably, VSMCs can be directly activated by circulating vasoactive substances, promoting coronary vasoconstriction. When endothelial function and coronary vasomotion are preserved, the interplay between endothelium and VSMCs results in coronary vasodilation; in case of endothelial dysfunction and/or VSMC hyperreactivity, the main effects are coronary vasoconstriction and tendency to CAS. The pathophysiology underlying endothelial dysfunction includes an enhanced synthesis of endothelin-1, which is a relevant vasoconstrictor, and an impaired release of NO [38,39,40].

### 3.2. Hyperreactivity of Smooth Muscle Cells

The hyperactivation of the RhoA/Rho-kinase pathway in muscular cells results in an increased sensitivity to vasoconstrictive stimuli by inducing the phosphorylation of myosin phosphatase [41,42,43]. Furthermore, the RhoA/Rho-kinase pathway is able to suppress endothelial NO synthase expression and activity, reducing the synthesis of NO and favoring the release of endothelin-1 [44].

These mechanisms contribute to coronary vasoconstriction, potentially impacting blood flow and cardiac function. Multiple mechanisms, embracing genetic and environmental features, contribute to CVD. Genetic polymorphisms include those implicated in endothelial function (aldehyde dehydrogenase * 2, NO synthase), oxidative stress (paraoxonase I) and inflammation (interleukin-6) [29,30,31]. It is important to acknowledge that several environmental stimuli might promote Rho-kinase activation, leading to coronary hypercontraction.

#### 3.2.1. Smoking Habit

Cigarette smoking is the major traditional cardiovascular risk factor implicated in CAS [45]. A smoking habit has been found to increase oxidative stress and to trigger the RhoA/Rho-kinase pathway [46].

#### 3.2.2. Chronic Low-Grade Inflammation

Chronic inflammation is a well-established trigger of coronary vasospasm. Several authors reported activated inflammatory pathways at systemic and coronary levels in patients with CAS [9]. Histopathological studies found pronounced adventitial pro-inflammatory milieu at the site of coronary vasospasm, with increased levels of eosinophils and neutrophils cells [47,48]. Intracoronary imaging tools, traditionally used to characterize coronary plaque phenotype and to guide percutaneous coronary interventions (PCI) [49], have contributed to understand CAS pathology: studies with intravascular ultrasound and optical coherence tomography revealed diffuse intimal thickening [50], as well as abundant macrophages and an exaggerated expression of vasa vasorum at spastic sites [51]. Importantly, by leveraging 18-fluorodeoxyglucose positron emission tomography/computed tomography, Ohyama et al. found a close link between the expansion of perivascular and pericoronary adipose tissue and the susceptibility to CVD [52]. Furthermore, patients with CAS have higher serum levels of inflammatory biomarkers compared to controls [53], as well as enhanced Rho-kinase activity of circulating leukocytes [54].

#### 3.2.3. Chronic Stress

CAS has been also linked to chronic stress and behavioral/psychological disorders [55,56]: chronic low-grade inflammation and hyperactive neuroendocrine/sympathetic system might mediate this association [57]. An elegant propensity-score matched analysis found that a history of anxiety confers a nearly five-high risk of developing CAS compared to controls [58]. From a mechanistic point of view, a sustained elevation of serum cortisol level is considered the key pathogenic mechanism by facilitating the activation of RhoA/Rho-kinase pathway. Importantly, this link is reversible, hinting at a possible target for the treatment of CAS [59]. Enhanced myelopoiesis in bone marrow and autonomic dysfunction may also predispose to CAS through reactive oxygen species (ROS) overproduction and impaired endothelial homeostasis [60].

#### 3.2.4. Air Pollution

Choi et al. were the first to note a positive association between the duration and levels of exposure of particulate matter 10 and the risk of CAS [61]. Later, Camilli et al. demonstrated that long-term exposure to pollutants—and to particulate matter 2.5 in particular—predicts a positive response to the ACh test and MINOCA as clinical presentation [62]. A putative pathogenetic role of low-grade inflammation and endothelial dysfunction, triggered by pollutants, has been postulated [63,64].

#### 3.2.5. Hypersensitivity

The Kounis syndrome is a potentially life-threatening cause of MI. Type 1 Kounis syndrome refers to CAS triggered by allergic or anaphylactic stimuli, such as poison ivy; bee stings; and reaction to shellfish, medications and contrast exposure [65]. The degranulation of activated mast cells initiates a complex cascade of inflammatory mediators, such as histamine, proteases and arachidonic acid products, leading to abnormal coronary vasoconstriction [66].

## 4. Invasive Provocative Testing

### 4.1. Biochemistry of Acetylcholine

ACh is an ester of acetic acid and choline [67], synthesized by the enzyme choline acetyltransferase, which converts free choline and acetyl-CoA into ACh. ACh is a main neurotransmitter of the parasympathetic system [68] and plays a crucial role in regulating cardiovascular system functions. ACh binds two families of receptors: muscarinic (M) receptors, that belong to the family of G-protein coupled receptors and consist of five subtypes (M1, M2, M3, M4, M5), with different signaling properties and function; and nicotinic (n) receptors, which are ligand-controlled ion channels composed of four different types of subunits [69].

ACh exerts pleiotropic effects on cardiovascular system. M receptors are the most relevant ACh receptors in the heart [69] and are widely expressed on the conduction system, cardiomyocytes and coronary arteries [70]. ACh regulates the electrical activity of the heart by binding M receptors (especially the M2-subtype) located in the sinoatrial and atrioventricular node and arterial baroceptors. In detail, ACh reduces the resting heart rate, facilitates the recovery of heart rate after physical activity, modulates the heart rate variability and preserves the baroreflex sensitivity. The M2 receptors subtype is the predominant isoform in the mammalian cardiomyocytes [69] and the ACh-induced activation of these receptors causes negative inotropic and dromotropic effects. Furthermore, ACh is implicated in cardioprotection and antagonism of ischemia-reperfusion injury: M3 and nα7 receptors have been found to mitigate the production of reactive oxygen species (ROS), preserve mitochondrial function and decrease the size of myocardial damage [69]. ACh is a major determinant of coronary artery tone modulation. Although M2, M3 and M5 receptors have been recognized [69], M3 receptors predominantly mediate the effect of ACh on endothelium and VSMCs [70]. M3 receptors are coupled with Gq proteins, promoting the activation of endothelial phosphoinositol-specific phospholipase C and the subsequent synthesis of inositol-1,4,5-triphosphate and diacylglycerol. This pathway causes the release of NO from functional endothelium [69], which is able to diffuse into VSMCs, favoring the synthesis of cyclic guanosine monophosphate, which drives coronary vasodilation. Conversely, in case of impaired endothelium, M3 receptors induce VSMCs intracellular calcium overload causing the activation of calmodulin-dependent kinase, which phosphorylates myosin light chain kinase, which in turn leads to coronary constriction [69].

### 4.2. Protocol

Given the transient nature of CAS, non-invasive tools are unlikely to detect it. Conversely, intracoronary administration of vasoactive stimuli elicits CAS in predisposed subjects with more than 90% sensitivity and 99% specificity [6,7,10]. Accordingly, invasive provocative tests are currently the gold standard for diagnosing vasomotor disorders.

Recently, the COVADIS Group [11] and the Japanese Circulation Society group [12] have proposed specific indications for invasive provocative testing. In detail, the ACh test is recommended in patients with clinical history suspicious of CAS, acute coronary syndrome presentation in the absence of a culprit lesion, unexplained resuscitated cardiac arrest, unexplained syncope preceded by chest pain and recurrent rest angina following angiographically successful coronary revascularization [11]. Moreover, most recent clinical practice guidelines have endorsed invasive provocative testing as part of the diagnostic work-up of patients with suspected INOCA/ANOCA and MINOCA [1,12,71,72,73]. In particular, the European Society of Cardiology guidelines advocate the use of coronary spasm provocation test in patients with NOCAD and a clinical picture of CAS with class of recommendation IIA and level of evidence B [71]. Furthermore, the Japanese Circulation Society recommends the coronary spasm provocation test in patients with MINOCA after excluding alternative diagnoses, such as plaque disruption and spontaneous coronary artery dissection, with class of recommendation IIB and level of evidence C [12]. 

ACh and ergonovine are the most used provocative agents. Ergonovine is a vasoactive substance that binds serotonergic receptors fostering VSMC contraction [74]. The diagnostic performance for the detection of CAS is high (91% sensitivity and 97%, specificity) [75]. Complications related to ergonovine use are rare [76]. The previously reported irreversible ergonovine-related complications were mainly contributed by intravenous high dose administration, while no death or myocardial infarction has been reported for three decades since the introduction of intracoronary low dose administration.

ACh is a parasympathetic nervous system transmitter that modulates vascular tone via muscarinic and nicotinic receptors. Under physiological circumstances, ACh facilitates the endothelial release of NO leading to vasodilatation; in case of endothelial dysfunction and consequent deficit of NO production, ACh targets VSMC resulting in coronary constriction [77].

The ACh provocative test is performed in the catheterization laboratory after baseline invasive coronary angiography. The discontinuation of calcium channel blockers (CCBs) and long-acting nitrates 48 h prior to the procedure is strongly recommended to avoid false-negative results. A continuous monitoring of patient symptoms and blood pressure, together with 12-lead electrocardiogram (ECG) and angiographic images, is required during the examination [12]. Different test protocols have been proposed, which differ with respect to ACh doses, injection speed and diagnostic criteria. Most frequently, a “stepwise approach”, consisting in the administration of increasing doses of intracoronary ACh (boluses of 20–50 and 100 mcg in the left coronary artery (LAD) and 20 and 50 mcg in the right coronary artery (RCA), injected 2–3 min apart) is used. The administration of a further dose of ACh of 200 mcg in the LCA and 80 mcg in the RCA in the absence of diagnostic criteria is questioned [78]. Sueda et al. addressed the safety and efficacy of the administration of 200 mcg of ACh in the LCA on top of the standard protocol in 88 patients. They found a nearly double rate of positive ACh test after administration of ACh 200 mcg compared to the standard protocol (40.9% vs. 19.3%) [78]. When the analysis was confined to 29 patients with rest angina, the rate of positive ACh test was almost three times higher (65.5% vs. 24.1%) after administration of ACh 200 mcg compared to the standard protocol. The specificity of this ACh spasm testing protocol was slightly lower (90%) compared to the standard protocol, due to the risk of pseudo-positive reactions. Notably, no major complications were recorded during administration of ACh 200 mcg. In summary, the intracoronary injection of 200 mcg of ACh in the LCA on top of the standard protocol (20–50–100 mcg) should be considered when clinical presentation is highly suspicious for CVD or when the administration of the first three doses (20–50–100 mcg) of ACh documented abnormal findings, such as ischemic ECG changes or new-onset chest pain or moderate coronary vasoconstriction, without fulfilling the criteria for epicardial or microvascular CAS [78]. 

**Figure 2:**
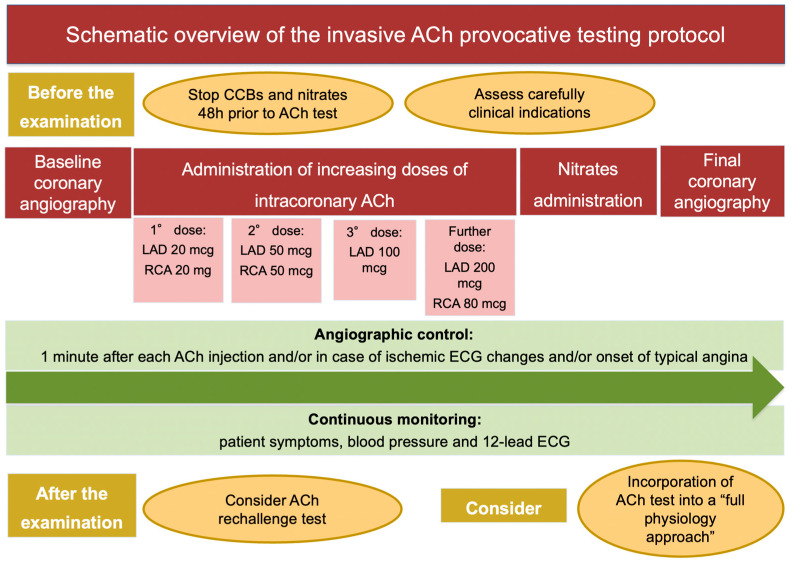
Schematic overview of the invasive ACh provocative testing protocol. Abbreviations: ACh: acetylcholine; CCBs: calcium channel blockers; ECG: electrocardiogram; LAD: left anterior descending artery; RCA: right coronary artery.

A 12-Lead ECG is recorded every 30 s, while coronary angiography is performed 1 min after each injection and/or in case of ischemic ECG changes and/or onset of typical angina. Intracoronary nitrates are the drug of choice to treat persistent coronary spasm. Recently, a German study tested the ability of a second dose of ACh after nitrates administration to refine the diagnosis of CAS: interestingly, the authors observed that epicardial and microvascular CAS coexist in 48% of patients and that they exhibit different response to nitrates: these are effective to prevent epicardial CAS in 85% and microvascular CAS in 20% [79]. The insertion of temporary pacemaker is debated. In this context, bradyarrhythmias are caused by transient reduction of blood flow to the conduction system of the heart [75]. An elegant histopathological study by Futami et al. revealed that the sinoatrial node was supplied by the RCA in 73% of patients and by both the LAD and the RCA is 23% of patients, while the atrioventricular nodal branch arose from the RCA in 80% of subjects and from both the RCA and the LCA in 10% of subjects [80]. Since the conduction system of the heart receives blood flow from the RCA in the large proportion of patients, the insertion of temporary pacemaker might be reasonable when intracoronary ACh is injected into the RCA [75], as indicated by the Japanese Circulation Society guidelines [12] (Table 1, Figure 2).

### 4.3. Endotypes

The following scenarios can be observed after a provocative test: (1) epicardial spasm: diagnosed when focal or diffuse epicardial coronary diameter reduction ≥90% is accompanied by reproduction of the patient’s symptoms and ischemic ECG shifts; (2) microvascular spasm: defined as the occurrence of typical angina and ischemic ECG changes without overt epicardial coronary constriction; (3) inconclusive response: in case of abnormal response in terms of chest pain, ECG changes or epicardial coronary constriction without diagnostic criteria for epicardial or microvascular spasm; (4) negative response: when no symptoms, ECG and angiographic modifications are observed [6,11].

The diagnosis of microvascular CAS is presumptive, since microvasculature cannot be visualized in vivo through coronary angiography. Microvascular CAS is universally diagnosed in case of reproduction of the patient’s symptoms and recognition of ischemic ECG changes (usually ST-segment depression), together with the absence of significant epicardial constriction (>90%) [11,12]. Notably, the diagnosis of microvascular CAS is often underestimated and masked by the concomitant presence of epicardial CAS. As mentioned before, Seitz et al. revealed that epicardial and microvascular CAS coexist in 48% of patients and confirmed that microvascular CAS is less responsive to nitrates [79]. In rare cases of refractory CAS despite administration of intracoronary nitrates, intravenous atropine should be administered [75]. The objective recognition of myocardial ischemia might be relevant to confirm the diagnosis of microvascular CAS, especially in case of concomitant epicardial CAS or when ECG changes and patient’s symptoms are difficult to interpret [90]. The measurement of lactate concentration in the coronary sinus during an ACh test, which is increased in case of ischemia due to enhanced anaerobic glycolysis, is recommended by the Japanese Circulation Society [12].

Patients with epicardial CAS tend to be more often male, with a great burden of traditional cardiovascular risk factors—in particular a smoking habit, along with a higher prevalence of diffuse non-obstructive coronary atherosclerosis—are more likely to present with MINOCA [84,91,92]. Conversely, patients with microvascular CAS tend to be more often female and to have a longer history of angina [33,34,35]. Notably, angina symptoms are highly subjective, and the presence and severity of angina is strongly influenced by behavioral and psychological features and neuroendocrine pathways [93].

Several reports found a clear-cut sex distribution of ACh-induced epicardial and microvascular CAS. Jansen et al. tested 264 patients with ANOCA: the prevalence of epicardial CAS was higher in men (63% vs. 42%), while women were more likely to exhibit microvascular CAS (40% vs. 29%) [94]. Montone et al. investigated the occurrence of CVD among 120 patients with ANOCA/MINOCA, showing a higher prevalence of microvascular CAS among women (81.4% vs. 18.6%) and of epicardial CAS among men (64.9% vs. 35.1%) [34]. Accordingly, in the study by Ohba et al., epicardial CAS occurred more frequently in men (70% vs. 49%), while women were more likely to exhibit microvascular CAS (21% vs. 3%; *p* < 0.0001) [95]. Conversely, other reports revealed a significantly higher prevalence of abnormal response to an ACh test in women: Aziz et al. found a higher rate of both epicardial CAS (28% vs. 23%; *p* < 0.05) and microvascular CAS (42% vs. 20%; *p* < 0.001) among women compared to men [33]. Similarly, both epicardial CAS (55% vs. 45%) and microvascular CAS (82% vs. 18%) occurred more frequently in women in the study by Ong et al. [96].

Microvascular spasm frequently coexists with CMD, which is highly prevalent among women [97]. Women were more likely to suffer from CMD. Several studies dealt with sex differences in coronary microvascular function [94,98,99,100]. The vast majority of reports found that women were more likely to have lower coronary flow reserve (CFR) values compared to men, while the index of microcirculatory resistance (IMR) values did not differ between sexes. The thermodilution-derived mean transit time was lower at rest in women and comparable between sexes during hyperemia. All these findings supported the hypothesis that women have elevated resting coronary blood flow, which, in turn, results in reduced capacity of coronary flow augmentation from rest to hyperemia [94,98,99,100]. Conversely, a computational fluid dynamics analysis by Taylor et al. noted higher coronary microvascular resistance in women compared with men [101]. Differences in enrolled population (the study by Taylor et al. [101] included a large proportion of patients with obstructive CAD, while the other studies included only patients with INOCA/ANOCA [94,98,99,100]), and invasive methods for assessing microvascular function might explain these differences. Furthermore, microvascular spasm is associated with a higher prevalence of diastolic dysfunction [102,103,104], a hallmark of heart failure with preserved ejection fraction, potentially suggesting a role in its pathogenesis [105,106]. Interestingly, patients with inconclusive response after an ACh test tend to share multiple clinical and angiographic features with those presenting with epicardial spasm [85].

It might be of clinical relevance to perform Invasive ACh provocative testing among healthy subjects. Whether the test could be positive at high intracoronary aCh doses even in normal people remains controversial [90]. Interestingly, Sueda et al. reported a positive ACh test in 9.1% of patients with non-ischemic heart disease. However, limited data are available as ethical concern precludes this analysis [107].

Finally, whether epicardial and microvascular spasm portend different prognosis is controversial. Lee et al. found similar outcomes between the two endotypes among 4644 patients with INOCA/ANOCA [85]; conversely, epicardial spasm was associated with worse prognosis at 1-year follow-up compared to microvascular spasm in a study by Montone et al. [7].

Coronary spasm can be also classified into focal CAS, characterized by a total or sub-total obstruction within the borders of one isolated coronary segment, and diffuse CAS, consisting of severe diffuse vasoconstriction of at least two adjacent coronary segments [17]. Diffuse CAS is associated to female sex and low prevalence of cardiovascular risk factors, while focal CAS occurs more frequently in men and is often associated with underlying atherosclerotic coronary lesions [82]. Intracoronary imaging studies revealed that atherosclerotic plaques in focal spastic segments exhibit often vulnerable features, such as high lipid content, macrophage infiltration, intraplaque neovessels and intracoronary thrombi, predisposing to destabilization [108]. Furthermore Teragawa et al. postulated that CAS contributes to thrombogenicity [109]. This finding might explain the worse prognosis observed in patients with focal compared to diffuse CAS [82].

### 4.4. Safety Profile

Several studies addressed the overall burden of complications occurring during an invasive ACh test. Pioneering works reported a rate of serious complications of 0.3–0.4% [76,110]. Later, experienced centers in Eastern countries showed a rate of life-threatening arrhythmias of 0.5–0.6%, with no deaths [18]. In a multicenter registry by the Japanese Coronary Spasm Association (1244 patients) the total arrhythmic burden was higher (3.2%). Potential explanations for this heterogeneity are twofold: the latter investigation included both sustained and non-sustained ventricular arrhythmias; furthermore, ACh was frequently injected into the RCA, that is often associated with diffuse spasm leading to ventricular tachycardia/fibrillation events [82]. 

Registries from Western countries reported lower rates of side effects during an invasive ACh test [81,86,87,111,112,113]. A sub-analysis of the WISE study showed that serious complications occurred in two women (0.7% of the total population) [81], while no major complications were noted in a seminal German study [6]. In keeping with these findings, a systematic review by Marrone et al. outlined a low number of global ACh-induced side effects (0.5%): the most frequent complications were arrhythmic events and patients with MINOCA have a significantly lower number of side effects when compared to those with INOCA [16]. A large meta-analysis comparing ACh and ergonovine reported a significantly higher rate of major (1.09% vs. 0.15%; *p* < 0.001) and minor complications (5.87% vs. 2.36%; *p* < 0.001) when the former was used as provocative stimulus [13]. Importantly, ACh-related death has never been described in the literature. More recently, Montone et al. observed an overall rate of complications of 9.1% during an ACh test. Bradyarrhythmias were the most frequently encountered complications (6.3%), followed by supraventricular tachyarrhythmias (2.5%); only two cases of life-threatening arrhythmias were reported [15,114]. Interestingly, the authors found that a previous history of paroxysmal atrial fibrillation, moderate-to-severe left ventricular diastolic dysfunction and higher QT dispersion at baseline ECG were the only predictors of procedural complications. These conditions share a latent electrical instability that might be precipitated by the administration of intracoronary ACh [115,116], which unevenly depolarizes ventricular epicardium and endocardium [116]. It is important to acknowledge that most ACh-related complications are transient due to the short half-life of ACh and can be promptly reversed through intracoronary nitrates administration. Some authors reported a risk of MI after intracoronary ACh administration [14,112]: in one case, it was possibly caused by a prolonged spasm of RCA, poorly responsive to intracoronary nitrates [14]; in the other one, the link between intracoronary ACh administration and the occurrence of MI was questionable [112]. Furthermore, two ischemic strokes were reported in the study by Tateishi et al. [14], likely related to coronary angiography rather than a risk carried by the ACh provocation test (Table 2).

### 4.5. Prognostic Relevance

In patients with CAS and stable clinical presentation, the prognosis is generally good and influenced by the concomitant presence of obstructive CAD [17,117]. The rate of all-cause death was 13% at 3 years in a Canadian study [118] and 7.2% at 7.4 years in a French registry [119]. However, ACh-provoked CAS was accompanied by obstructive CAD in a sizeable proportion of enrolled patients. Conversely, Figueras et al. found a very low rate (7.0% at 12-years follow-up) of cardiac mortality among 273 patients presenting with CAS and INOCA/ANOCA [120]. Large Asian registries have drawn similar conclusions [83,88]. Shimokawa et al., investigating 1244 Japanese patients with CVD, reported a rate of 1.3% of all-cause death [121], while the 2-year incidence of cardiac death was 0.9% in a Korean study [122]. Lastly, major adverse cardiovascular events (MACE) occurred in 72 of 4644 (1.6%) patients with INOCA/ANOCA in a study by Lee et al. [83].

In patients admitted for MINOCA, the diagnosis of CAS confers a higher risk of MACE, compared to those with a negative ACh test. In 986 patients with a positive ACh test and NOCAD, presenting with MINOCA (versus INOCA/ANOCA) portended a nearly double risk of MACE, driven by a higher occurrence of cardiac death and recurrent MI, at a median follow-up of 4.4 years [123]. Similarly, Montone et al., enrolling 80 patients with MINOCA and suspected CVD, showed that a positive ACh test led to a significantly higher occurrence of death from any cause, cardiac death and readmission for acute coronary syndrome compared to negative response [7]. In contrast, two studies showed good outcome among patients with CAS and MINOCA [22,124]. However, most patients enrolled in these studies presented with unstable angina, suggesting a more favorable clinical profile that in turn could translate into a lower risk of future adverse events.

This evidence supports the hypothesis that patients presenting with MINOCA and a positive ACh test represent a high-risk subset of patients with a more aggressive phenotype of CAS characterized by more severe coronary vasoconstriction and/or more prolonged ischemia time and/or more extended spastic coronary segments. CAS-induced myocardial necrosis causes a chronic low-grade inflammatory response that might perpetuate the myocardial damage leading to worse long-term outcome [125]. In keeping with this hypothesis, multi-vessel CAS, elevated C-reactive protein levels and out-of-hospital cardiac arrest predicted a worse prognosis among 97,280 patients hospitalized for CAS [126].

Furthermore, patients with CVD are at increased risk for life-threatening arrhythmic events and cardiac arrests [127]. Recurrent episodes of CAS might promote electrophysiological disorders, such as early repolarization, pattern of horizontal/descending ST segment elevation and QT dispersion and might cause a reentry circuit surrounding a myocardial scar, which ultimately predispose to sudden cardiac death [115,128,129]. Patients who survived after out-of-hospital cardiac arrest experienced a long-term worse prognosis compared to without aborted sudden cardiac death, which was mainly driven by higher rates of cardiac death and death from any cause [130]. Therefore, the implantation of an automatic cardiac defibrillator might be lifesaving for cardiac arrest survivors [131]. 

It is worth noting that CVD significantly worsen quality of life, often resulting in repeated coronary angiographies, showing in most cases no atherosclerotic changes compared to baseline examination [132]. In detail, the rate of repeated coronary angiographies is up to 10% in patients with a positive ACh test and about twice compared to patients without CAS [85]. In keeping with these findings, in the CASPAR study [22], half of patients experienced recurrent angina and Montone et al. reported a worse angina status, as assessed by the Seattle Angina Questionnaire score, in patients with a positive ACh test compared to those with a negative response [15].

### 4.6. Invasive Acetylcholine Evaluation and Its Interplay with Other Functional Tests

In recent decades, a growing body of evidence has demonstrated that approximately one-fifth of patients may manifest concurrent vasomotor disorders alongside CMD [133]. Notably, these alterations can also coexist with CAD. These findings advocate for the integration of the ACh test into a more comprehensive functional assessment of the coronary tree.

Recently, leading experts in the field have proposed a “full physiology approach”, delineating a step-by-step protocol addressing each component of the coronary circulation, including epicardial; microvascular; and ultimately, vasomotor function [134]. In detail, the initial step involves the functional evaluation of the epicardial and microvascular domains through adenosine infusion. Adenosine explores endothelium-independent vasodilation, providing insights into the microvascular vessel’s ability to increase flow under maximum hyperemia. This helps identify hemodynamically relevant stenoses (Fractional Flow Reserve (FFR) < 0.80), impaired CFR (<2.5), and elevated IMR (>25) addressing the fixed component of coronary tree and revealing “structural impairment” in various domains. The second step involves ACh infusion to assess the physiological vasodilator response mediated by the endothelium. Within the COVADIS criteria framework, the conventional diagnosis of epicardial spasm currently relies on visually appraising epicardial narrowing during angiography (exceeding 90%). However, the visual evaluation of coronary stenosis has demonstrated considerable interobserver variability. Moreover, angiographic stenosis assessment often fails to adequately consider the hemodynamic repercussions of coronary narrowing, potentially leading to an underestimation of functionally significant epicardial spasm.

Presently, microvascular spasm is often considered a “negative” or “presumed” diagnosis when Ach-induced ischemia (manifesting as angina onset and ischemic ECG changes) occurs without evidence of epicardial spasm. The concept of “microvascular spasm” derived from earlier studies that noted increased lactate levels in the coronary sinus and/or the occurrence of a slow flow phenomenon during ACh infusion, all in the absence of epicardial spasm [95,135,136,137]. Notably, these studies did not take into account information pertaining to microvascular flow and resistance. Consequently, to enhance the diagnostic accuracy of the ACh test and to gain deeper insights into the pathological mechanisms of myocardial ischemia, some authors recommend the integration of thermodilution/pressure wire use to assess hemodynamic parameters during ACh infusion. This encompasses pressure recordings (Pd/Pa) and flow/resistance parameters (CFRACh, IMRACh). In the epicardial domain, a reduced Pd/Pa during ACh infusion, as detected by the pressure wire within the coronary artery, could lend support to epicardial CAS diagnosis. Simultaneously, a functional assessment using bolus thermodilution techniques in the microvascular domain could confirm the diagnosis of microvascular spasm by revealing an increase in microvascular resistance (IMRACh) during ACh infusion. Studies conducted by Nardone et al. have demonstrated that various pharmacologic agents (adenosine, ACh, etc.) may assist in interrogating different microvascular control mechanisms, thereby supporting the utilization of both tests [138]. Additionally, Miner and colleagues have shown that patients experiencing ACh-induced chest pain exhibit a higher prevalence of epicardial endothelial dysfunction and pathological high microvascular flow at rest [139]. The ongoing multicenter registry, “Searching a New Acetylcholine sPasm dEfinition” (SNAPE), is actively assessing the effectiveness of a “wire-based assessment” in reclassifying epicardial and microvascular spasms according to COVADIS criteria. This registry proposes new “hemodynamic criteria” that aim to contribute to a better understanding and definition of the pathological mechanisms underlying vasomotor disorders. 

## 5. Therapy

The therapeutic approach of CVD is challenging: the underlying pathophysiology is heterogeneous and limited evidence arising from randomized trials is available. The optimal management of patients with CAS hinges on some basic principles: (1) the invasive diagnosis of CVD is key to implement targeted therapies; (2) available treatments are effective in reducing MACE and in improving quality of life; (3) targeted therapy should be started as early as possible: most life-threatening complications occur within the first months after symptom onset [140]; (4) a meticulous follow-up is necessary: medication should be carefully uptitrated to ensure clinical benefits while minimizing side effects; (5) patient adherence to therapy is crucial: the abrupt discontinuation of therapy increases the risk of rebound phenomenon leading to more severe CAS and worse clinical outcome [141].

### 5.1. Calcium-Channel Blockers

CCBs are the mainstay therapy for CAS. CCBs inhibit voltage-dependent L-type calcium channel in VSMCs modulating the actin-myosin interaction and causing artery dilatation together with negative inotropic and chronotropic effects. CCBs are categorized into: dihydropyridine (DHP), such as nifedipine, amlodipine and benidipine; and non-DHP, such as verapamil and diltiazem. While non-DHP CCBs show low selectivity for vascular and cardiac receptors, DHP CCBs specifically target arterial districts [142]. 

Both DHP and non-DHP CCBs have been proven to reduce the frequency and intensity of angina episodes along with the occurrence of MI and life-threatening arrhythmias [143,144,145,146]. A recent meta-analysis found that benidipine, a second-generation of DHP CCB with high affinity for the coronary vessels, might be the most effective CCB for improving both symptoms control and outcomes [147]. However, the lack of randomized controlled trials precludes drawing definitive conclusions.

The elegant EDIT-CMD (Efficacy of Diltiazem to Improve Coronary Microvascular Dysfunction: A Randomized Clinical Trial) trial enrolled 73 patients with ANOCA and a diagnosis of coronary vasospasm and/or microvascular dysfunction: 6-week therapy with diltiazem, although effective at preventing epicardial CAS in most patients, failed to demonstrate benefits in terms of coronary microvascular function and quality of life compared to placebo, suggesting that CCBs are ineffective when ANOCA is driven by mechanisms other than CAS [148]. Ankle oedema is the most common side effect of CCBs, followed by constipation. Notably, CAS may occur even with high doses of CCBs, and combined therapies are necessary in more than half of patients [149].

### 5.2. Nitrates

Nitrates should be considered as a second-line strategy reserved to patients who remain symptomatic despite CCBs therapy [1]. Mechanisms of action of nitrates are multifaceted: they mitigate the activity of VSMC Rho-kinase and foster the endothelial release of NO. Short-acting nitrates should be preferred since they effectively suppress ongoing angina attacks [150]. Contrariwise, prolonged long-term nitrates therapy might increase the risk of MACE [151]. Potentially, this results from nitrate-related overproduction of ROS, with consequent tachyphylaxis and endothelial dysfunction [152]. The most relevant side effects include headache, orthostatic hypotension and flushing [150].

### 5.3. Nicorandil

Nicorandil exerts vasodilator effects on both coronary epicardial and microvascular districts, promoting the endothelial release of NO and the activation of K^+^_ATP_ channels and ultimately causing the inhibition of voltage-dependent L-type calcium channels [153]. A landmark study documented the capability of nicorandil to reduce the frequency of angina attacks in up to 75% of patients [153]. However, subsequent studies failed to demonstrate prognostic benefits of nicorandil [153,154]. Current guidelines support nicorandil use in case of refractory angina [1]. Headache, dizziness and hypotension are the most common side effects, while mucosal and eye ulcerations are the most feared complications. Importantly, long-term therapy is not associated with tachyphylaxis [154].

### 5.4. Statins

Statins exert pleiotropic effects that might be advantageous in the context of CVD: they prevent the activation of VSMC Rho-kinase, downregulate inflammatory pathways and the endothelial production of ROS. The addiction of Fluvastatin to CCBs therapy was able to halve the rate of ACh-elicited CAS at a 6-month follow-up in a randomized trial enclosing 64 patients with NOCAD and a positive response to the ACh test [155]. Accordingly, Piao et al. found an inverse relationship between statin therapy and the occurrence of recurrent MI among patients with CAS presenting with MINOCA [156]. However, recent studies failed to show prognostic benefits of statins [157,158].

### 5.5. Fasudil

Since it specifically targets the RhoA/Rho-kinase pathway, fasudil has been proposed as a further therapeutic approach for patients with CAS [39]. Masumoto et al. tested the efficacy of fasudil pretreatment, compared to intracoronary saline, in preventing recurrent CAS among a cohort of 20 patients with a positive ACh test. After second dose of ACh, fasudil significantly reduced coronary constriction, as well as the occurrence of chest pain and ischemic ECG changes [159]. Furthermore, fasudil pretreatment has been shown to suppress myocardial ischemia—as proven by improved lactate extraction ratio—in approximately two-thirds of patients with microvascular angina (MVA) [160]. However, to date, fasudil is not available in Western countries.

### 5.6. Endothelin-1 Inhibitors (ET-1)

ET-1 is a fascinating therapeutic target, since this peptide is involved in several pathways related to endothelial homeostasis and VSMCs tone regulation. Genetic polymorphisms of ET-1 facilitate the development of CAS [30] and a landmark study by Toyo-oka et al. showed high levels of endothelin-1 in the coronary microenvironment of patients with CAS [161]. However, the recent placebo-controlled randomized VERA trial failed to demonstrate benefits from Macitentan, a potent inhibitor of the ET-1 receptor, on anginal burden among 28 patients with Ach-induced CAS and >3 anginal attacks per week [162].

### 5.7. Cilostazol

Cilostazol is a phosphodiesterase type III inhibitor with vasodilator, antiplatelet and anti-inflammatory activities [163]. Watanabe et al. reported an improvement in coronary microvascular function after cilostazol therapy [164]. Moreover, cilostazol was able to mitigate angina frequency and severity in the placebo-controlled randomized STELLA trial (Study to evaluate the Efficacy and safety of Pletaal ciLostazoL in subjects with vAsospastic angina) [165].

### 5.8. Molsidomine

Molsidomine is a long-acting vasodilator able to elicit NO release via the metabolite SIN-1 [166]. A small single center experience demonstrated that Molsidomine is able to suppress CAS in 80% of patients with angina and coronary spasm [167].

### 5.9. Coronary Revascularization and Sympathetic Denervation

Revascularization, with either PCI [168] or coronary artery bypass graft [169], might be considered in case of refractory spasm despite appropriate medical therapy. While PCI is effective for treating CAS at the site of focal atherosclerotic plaque, this strategy may be unsuccessful in case of diffuse spasm [170]. Furthermore, coronary stents might further exacerbate the susceptibility to CAS [171].

Sympathetic denervation has also been anecdotally reported to suppress refractory CAS [172]; however, data is scant. 

### 5.10. Lifestyle Recommendation and Triggers Avoidance

**Figure 3:**
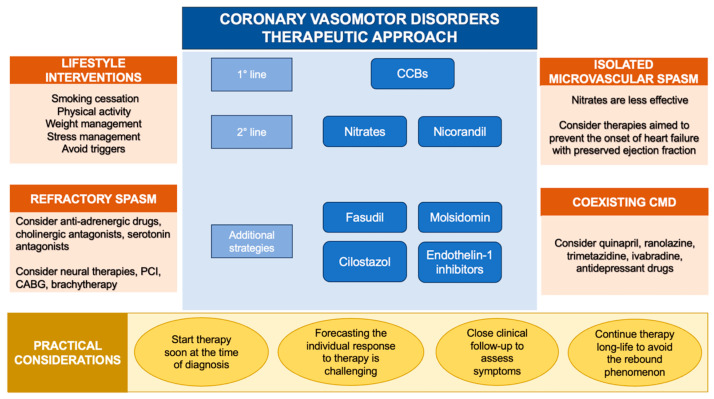
Therapeutic approach to coronary vasomotor disorders. Abbreviations: CABG: coronary artery bypass grafting; CCBs: calcium channel blockers; CMD: coronary microvascular dysfunction; PCI: percutaneous coronary intervention.

Avoiding exposure to specific triggers (medications, abuse drugs and allergenic substances) could be lifesaving when CAS arises in the context of identifiable vasoconstrictive stimuli [63]. When medications, such as chemotherapeutic drugs, are crucial for the treatment of specific diseases and cannot be discontinued, the concomitant administration of CCBs might be a reasonable strategy [131]. Furthermore, specific lifestyles (smoking habit, alcohol consumption, chronic stress and behavioral disorders) trigger CAS in predisposed subjects [45,55,56]. As a result, long-term lifestyle modifications and the implementation of new broader therapeutic strategies, including psychological support, should be encouraged [173] (Figure 3).

## 6. Follow-Up

A meticulous follow-up is of paramount importance for managing patients with CVD. The main reason is that forecasting individual patient response to therapy is challenging. Medications should be carefully up titrated to address angina symptoms while avoiding side effects. For patients who remain symptomatic, combination therapies are required [11]. 

According to some authors, patients with documented CAS should undergo ECG-Holter monitoring to detect life-threatening ventricular arrhythmias, which can be triggered by silent CAS [174]. Non-invasive provocative testing, such as hyperventilation and cold pressor testing, are seldom performed to reassess coronary vasoreactivity, due to their low diagnostic accuracy [12].

## 7. Challenging Clinical Scenarios

### 7.1. Isolated Microvascular Spasm

Isolated microvascular spasm is a clinical entity characterized by diffuse constriction of the small vessels that modulate myocardial blood flow. Current guidelines recommend a similar therapeutic approach for patients with epicardial and microvascular CAS [1,11,12]. However, recent evidence indicates that patients with microvascular CAS are more likely to suffer from psychological disorders [175] and experience higher rates of recurrent angina along with worse quality of life when compared to those with epicardial CAS [34]. This evidence suggests a distinct pathophysiology underlying the two endotypes of CAS and underlines that available medications are less effective for treating microvascular than epicardial spasm [176]. Accordingly, Seitz et al. demonstrated by Ach rechallenge test that nitroglycerin is able to prevent recurrent epicardial CAS in 85% of patients and recurrent microvascular CAS only in one-fifth of patients [79]. Thus, the search for novel pathophysiological determinants of small vessels vasoconstriction is of clinical relevance, and should be accompanied by the implementation of a multidisciplinary therapeutic approach aimed at managing precipitating factors [177].

### 7.2. Coexisting Microvascular and Vasospastic Angina

There is growing evidence that the association of CVD and CMD might portend worse quality of life when compared to isolated conditions [134]. A phenotype characterization if INOCA/ANOCA patients is crucial. The landmark CorMicA (Coronary Microvascular Angina) trial showed that a targeted medical therapy is able to improve angina burden and quality of life at 1 year compared to empirical treatment [87]. Furthermore, a sub-analysis of the CorMicA study documented the benefits of a tailored therapy in terms of cost-effective use of resources, mainly driven by a reduction of recurrent hospitalization for repeat coronary angiography procedures [178].

The optimal therapeutic strategy for patients with coexisting CVD and CMD is challenging. A combination of medications should be administered [179]: CCBs are the drug of choice, and the association of ACE-inhibitors and statins is often indicated. In case of inadequate control of symptoms, the addition of nicorandil, ranolazine and trimetazidine could be an option [180]. Beta-blockers should be avoided because they may exacerbate CAS [181]. Nitrates, especially when MVA is the leading underlying pathophysiology, should be used with caution since they may promote a stealing effect, potentially worsening symptoms [182]. 

### 7.3. Patients with Myocardial Bridging

Myocardial bridging (MB) is a common congenital coronary anomaly, in which a portion of an epicardial coronary artery takes an intramuscular course resulting in a dynamic compression of the tunneled segment during the systole [183]. While MB is an incidental finding in most patients, sometimes the long-term compression–relaxation effect of MB on the coronary artery might promote endothelial dysfunction predisposing to CAS [184]. Additionally, studies have shown a decrease in the expression of endothelial NO synthase in the MB segment when compared to the proximal and distal segments [185]. 

Recently, Montone et al. demonstrated that the presence of MB predicts MINOCA as clinical presentation and a positive response to an ACh test. Importantly, the subset of patients with MB and a concomitant positive ACh test experienced worse cardiovascular outcome at a mid-term follow-up [186]. Although beta-blockers are the first-line therapy for MB, they should not be prescribed or should be withdrawn when the latter is associated with CAS [187].

### 7.4. Patients with Obstructive CAD Undergoing PCI

Up to 40% of patients experience recurrent/persistent angina at 1 year after successful PCI [188]. Structural (diffuse atherosclerosis, CAD progression, in-stent restenosis and thrombosis, MB) and functional (CVD and CMD) mechanisms are responsible for the occurrence of angina after PCI [189]. A large study by Ong et al. documented a positive ACh test in nearly two-thirds of patients with angina and no evidence of PCI failure (49% epicardial CAS, 17% microvascular CAS) [190]. Thus, an ACh test could identify CVD in a large proportion of patients with recurrent/persistent angina after successful PCI, helping to implement a tailored therapy and avoiding further unnecessary coronary angiographies and PCI [191].

## 8. Future Perspectives

From a diagnostic perspective, the integration of an invasive ACh test into everyday clinical practice, according to COVADIS [11] and Japanese Circulation Society [12] indications, is imperative because the missed diagnosis of CAS results in poor quality of life and increased risk of MACE [6,7,83,88,118,119,120,121,122,123,124,125]. The results of the SNAPE registry will add new insights into the hidden mechanisms of vasomotor disorders thanks to the assessment of functional parameters during an ACh test. 

From a therapeutic point of view, no study has addressed the effect of a physiology-based therapeutic approach on clinical hard endpoints. The ongoing “PROgnostic Value of Precision Medicine in Patients With Myocardial Infarction and Non-obStructive Coronary artEries” (PROMISE) trial (ClinicalTrials.gov: NCT05122780) [192], INOCA-IT Multicenter Registry (ClinicalTrials.gov: NCT05164640) and PRIZE trial (ClinicalTrials.gov: NCT04097314) [193] will help to close this knowledge gap. 

## 9. Conclusions

CVDs originate from the complex interplay between genetic predisposition and environmental stimuli and are associated to an elevated risk of future MACE. The invasive, provocative ACh test is the gold standard tool to diagnose epicardial and microvascular CAS according to COVADIS criteria. CCBs are the mainstay therapy for patients with CAS. Several pharmacological or interventional therapeutic options, in addition to CCBs, should be considered for patients who remain symptomatic despite high doses CCBs.

Novel therapies targeting mechanistic pathways need to be implemented in order to devise a personalized therapy of different endotypes of CVD.

## Figures and Tables

**Table 1:** Overview of different protocols of ACh provocative spasm testing.

First Author, Year [Ref.]	Population	Clinical Presentation	Time of ACh Injection	ACh Doses (μg)	Threshold of Coronary Constriction	Epicardial CAS (%)	Microvascular CAS (%)
Wei, J., 2012 [81]	293	INOCA	3 min	LCA: 0.182–1.82–18.2 μg/mL RCA: NA	>50–70%	7.3	NA
Takagi, Y., 2013 [82]	713	Suspected VSA	20 s	LCA: 20–50–100 RCA: 20–50	>90%	100	NA
Sato, K., 2013 [17]	873	Suspected VSA	20 s	LCA: 20–50–100 RCA: 20–50	>90%	49.6	NA
Ong, P., 2014 [6]	847	INOCA/MINOCA	3 min	LCA: 2–20–100–200 RCA: 80	>75%	33.4	24.2
Nakayama, N., 2014 [24]	211	MINOCA	NA	LCA: 20–50–100 RCA: 20–50	>90%	79.2	NA
Choi, W.G., 2016 [83]	4305	INOCA	60 s	LCA: 20–50–100 RCA: -	>70%	77.7	NA
Di Fiore, D.P., 2015 [84]	183	ANOCA	20 s	LCA: 25–50 RCA: 20	>90%	44.3	32.0
Lee, E.M., 2017 [85]	4644	INOCA	NA	LCA: 20–50–100 RCA: -	>75%	13.9	29.5
Tateishi, K., 2018 [14]	529	INOCA/MINOCA	20 s	LCA: 20–50–100 RCA: 20–50	NA	48.8	NA
Deyama, J., 2018 [23]	437	Poat-MI	30 s	LCA: 50–100 RCA: 50	>90%	44.6	NA
Probst, S., 2018 [86]	180	INOCA/MINOCA	3 min	LCA: 2–20–100–200 RCA: 80	>90%	26.1	42.2
Ford, T.J., 2018 [87]	145	ANOCA	20 s	LCA: 100 RCA: 50	>90%	37.1	32.5
Sueda, S., 2020 [88]	1810	Suspected VSA	20 s	LCA: 20–50–100–200 RCA: 20–50–80	>90%	59.3	NA
Montone, R.A., 2021 [15]	317	INOCA/MINOCA	3 min	LCA: 20–50–100–200 RCA: 20–50	>90%	37.7	21.3
Bil, J., 2021 [89]	211	Suspected VSA	3 min	LCA: 25–50–100 RCA: 25–50–75	>90%	46.9	34.1

Legend to table: ACh: acetylcholine; ANOCA: angina with no-obstructive coronary artery disease; CAS: coronary artery spasm; INOCA: ischemia with no-obstructive coronary artery disease; LCA: left coronary artery; MINOCA: myocardial infarction with no-obstructive coronary artery disease; NA: not available; RCA: right coronary artery; VSA: vasospastic angina.

**Table 2:** Summary of the main studies (including >100 patients) on adverse events related to ACh provocative testing complications.

First Author, Year [Ref.]	Type of Study	Population	Clinical Presentation	Minor Adverse Events [n (%)]	Major Adverse Events [n (%)]
Encore, I., 2003 [111]	RCT	343	CCS	NA	2 (0.6) hemodynamic instability events requiring resuscitation
Lüscher, T.F., 2009 [112]	RCT	454	CCS	NA	2 (0.4): 1 hemodynamic instability event requiring resuscitation, 1 MI
Wei, J., 2012 [81]	Observational	293	INOCA	0 (0.0)	0 (0.0)
Takagi, Y., 2013 [82]	Observational	713	Suspected VSA	34 (4.8) 1 PVC 29 bradyarrhythmias	35 (4.9) VT/VF
Sato, K., 2013 [17]	Observational	873	Suspected VSA	NA	9 (1.0) life-threatening arrhythmias
Ong, P., 2014 [6]	Observational	847	INOCA/MINOCA	8 (0.9) 1 TV 1 AF 5 bradyarrhythmias	0 (0.0)
Choi, W.G., 2016 [83]	Observational	4305	INOCA	1310 (30.4) bradyarrhythmias	0 (0.0)
Di Fiore, D.P., 2015 [84]	Observational	183	ANOCA	5 (2.7) 1 bradyarrhythmia 4 AF	0 (0.0)
Lee, E.M., 2017 [85]	Observational	4644	INOCA	1334 (28.7) 1328 bradyarrhythmias 6 AF	0 (0.0)
Tateishi, K., 2018 [14]	Observational	529	INOCA/MINOCA	58 (11.0) 4 non-sustained VT 54 AF	7 (1.3): 1 VT, 1 MI, 3 CS, 2 strokes
Probst, S., 2018 [86]	Observational	180	INOCA/MINOCA	28 (15.5) 25 bradyarrhythmias 3 FAP	0 (0.0)
Ford, T.J., 2018 [87]	RCT	145	ANOCA	9 (6.0) 1 persistent AF 8 paroxysmal AF	0 (0.0)
Sueda, S., 2020 [88]	Observational	1810	Suspected VSA	315 (7.4) AF	41 (2.26): 29 VT/VF, 11 Shock, 1 cardiac tamponade
Pargaonkar, V.S., 2020 [27]	Observational	277	ANOCA	31 (11.3) 28 bradyarrhythmias 3 AF	0 (0.0)
Montone, R.A., 2021 [15]	Observational	317	INOCA/MINOCA	28 (8.8) 20 bradyarrhythmias 8 AF/SVT	2 (0.6) VT/VF
Bil, J., 2021 [89]	Observational	211	Suspected VSA	2 (0.9) AF 16 (7.6) bradyarrhythmias	0 (0.0)

Legend to table: ACh: acetylcholine; AF: atrial fibrillation; ANOCA: angina with no-obstructive coronary artery disease; CCS: chronic coronary syndrome; CS: cardiogenic shock; INOCA: ischemia with no-obstructive coronary artery disease; MI: myocardial infarction; MINOCA: myocardial infarction with no-obstructive coronary artery disease; NA: not available; PVC: premature ventricular contraction; RCT: randomized controlled trial; SVT: supraventricular tachycardia; VF: ventricular fibrillation; VSA: vasospastic angina; VT: ventricular tachycardia.

## Data Availability

No new data were created or analyzed in this study. Data sharing is not applicable to this article.
